# Electrophysiological Studies in Thyroid Associated Orbitopathy: A Systematic Review

**DOI:** 10.1038/s41598-017-11998-0

**Published:** 2017-09-21

**Authors:** Tiara W. U. Iao, Shi Song Rong, An Ni Ling, Mårten E. Brelén, Alvin Lerrmann Young, Kelvin K. L. Chong

**Affiliations:** 1 Department of Ophthalmology and Visual Sciences, The Chinese University of Hong Kong, Hong Kong, China; 2 Department of Ophthalmology and Visual Sciences, The Prince of Wales Hospital, Hong Kong, China; 3 Present Address: Harvard Medical School, and Massachusetts Eye and Ear Infirmary, Boston, Massachusetts USA

## Abstract

Dysthyroid optic neuropathy (DON) is the commonest cause of blindness in thyroid associated orbitopathy (TAO). While diagnosis remains clinical, objective tests for eyes with early or equivocal findings are lacking. Various electrophysiological studies (EPS) have been reported, yet the types and parameters useful for DON remain inconclusive. We performed a systematic literature search in MEDLINE, EMBASE and the Cochrane databases via the OVID platform up to August 20, 2017. 437 records were identified for screening and 16 original studies (1327 eyes, 787 patients) were eligible for review. Pattern visual evoked potential (pVEP) was the most frequently studied EPS. Eyes of TAO patients with DON showed delayed P100 latencies, decreased P100 amplitudes or delayed N75 latencies during pVEP, compared to those without or healthy controls. Due to study heterogeneity, no quantitative analysis was possible. This review highlights the most common type (pVEP) and useful parameters (P100 latency and amplitude) of EPS, and supports further research on them using standardized testing conditions.

## Introduction

Dysthyroid optic neuropathy (DON) is the commonest blinding complication affecting 4–8% of patients with thyroid associated orbitopathy (TAO), with an estimated annual incidence of 0.6–1.3 cases per 100,000 population^1,2^. While exact mechanisms of DON remain elusive, apical compression by enlarged extraocular muscles and/or fat (crowding)^3,4^, ischemia due to increased retrobulbar pressure, mechanical stretch due to proptosis and perineural inflammation have been proposed^5^. Empirical treatments including surgical apical decompression, systemic steroids and orbital radiotherapy are often effective to restore vision. It is thus imperative to confirm diagnosis early to avoid irreversible visual loss and unnecessary treatments in alternative causes^5^. Ancillary tests, for example optical coherence tomography^6^, orbital imaging^7^ and electrophysiological studies (EPS), including visual evoked potential (VEP) and electroretinogram (ERG) were attempts to objectively assess the presence, predict the development and correlate with the severity of DON^8–23^. However, methodologies and results were heterogeneous across studies. In this systematic review, we studied published reports on EPS in DON.

## Results

### Characteristics of included studies

**Figure 1:**
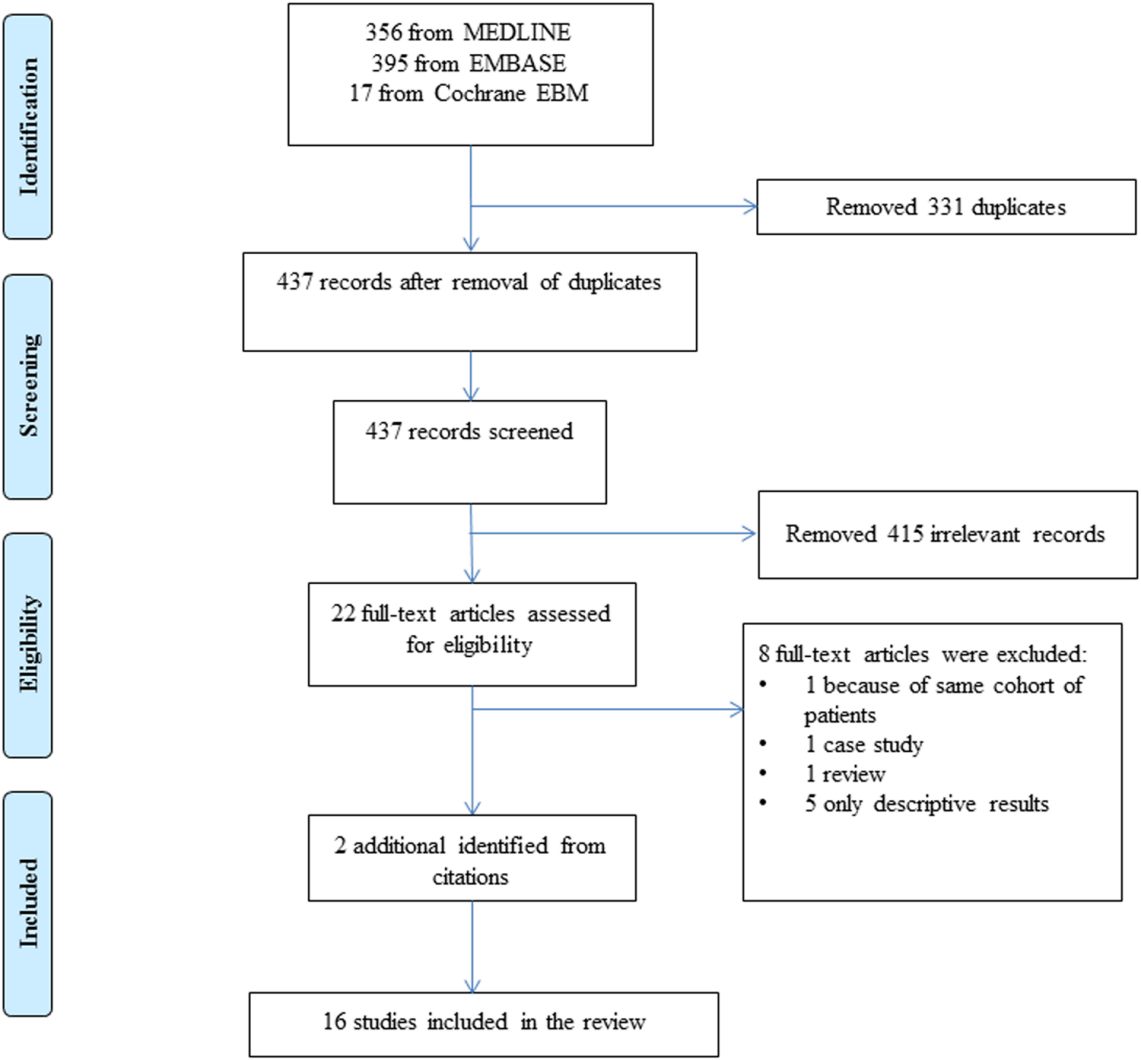
Flowchart of the literature search and study selection process.

Our search yielded 768 reports from databases. After removing 331 duplicated records, we studied 437 publications. Among them 415 studies were found to be irrelevant according to our eligibility criteria (see Methods below). For the remaining 22 studies, 8 reports were excluded: 1 report on a duplicated study population^24^, 1 case report^25^, 1 review article^26^, and 5 studies with irrelevant or insufficient results^1,27–30^. 2 additional studies were identified from manual search of references^8,9^. 16 studies were finally included for the systematic review (Fig. 1)^8–23^. No clinical trial was identified.

The pooled sample included 787 patients (1,327 eyes). The age of patients with DON ranged from 14 to 77 years old^13^. VEP was used in 14 studies^8–13,15–22^. 3 studies tested pERG^12,14,23^. No study was found on flash or multifocal ERG (Table 1).

**Table 1:** Characteristics of included studies in the systematic review.

	Author (year)	Country/region	Study Design	EPS tested	ISCEV	Sample size	TAO Age range (mean)	DON Age range (mean)	Subgroups	Outcomes
1	Wijngaarde *et al*.^8^	The Netherlands	Prospective case series	pVEP	✗✗	53	n.a.	—	TAO/Control	Correlation between P100 (latency) and VA
2	Setala *et al*.^9^	Finland	Prospective case series	fVEP	✗	31	28–66	—	TAO	The differences in N60 & P120 (amplitude & latency) before and after TAO treatment
3	Shawkat *et al*.^10^	England	Prospective case-control study	pVEP	✗	20	37–62 (47.3)	37–62 (47.3)	DON/TAO/Control	The differences in P100 (amplitude & latency) among DON, TAO and control
4	Tsaloumas *et al*.^11^	UK	Retrospective case series	fVEP, pVEP	✗	43	23–68 (45.1)	26–73 (49.1)	DON/TAO/Control	The differences in P2 (amplitude & latency) among DON, TAO and control; The differences in P2 (amplitude & latency) before and after DON treatment
5	Spadea *et al*.^12^	Italy	Prospective case series	pVEP, pERG	✗	49	(57.2)	—	TAO/Control	The differences in P100 (amplitude & latency) between TAO and control
6	Salvi *et al*.^13^	Italy	Retrospective case series	pVEP	✗	117	14–77 (45.3)	—	TAO/Control	The differences in P100 (amplitude & latency) between TAO and control
7	Genovesi-Ebert *et al*.^14^	Italy	Prospective case series	pERG	✗	44	(51.9)	—	TAO/Control	The significant difference in amplitude between TAO and control
8	Rutecka-Debniak *et al*.^15^	Poland	Prospective case series	pVEP	✗	110	18–74	18–74	DON/TAO	The differences in N75 & P100 (latencies) between DON and TAO; The differences in N75 & P100 (latencies) before and after DON & TAO treatment
9	Acaroglu *et al*.^16^	Turkey	Prospective case series	pVEP	✗	31	20–65 (41.7)	—	TAO/Control	The difference in P100 (latency) between TAO and control; Correlation between P100 (latency) and CAS
10	Ambrosio *et al*.^17^	Italy	Prospective case-control study	pVEP	✗	63	(36.3)	(42.5)	DON/TAO/Control	The differences in P100 (amplitude & latency) between DON and control
11	Pawlowski *et al*.^18^	Poland	Prospective case series	pVEP	✓2004^49^	27	35.6 ± 11.3	—	TAO/Control	The differences in N75 (latency) & P100 (amplitude & latency) between TAO and control; Correlation between N75 & P100 (latencies) and IOP & degree of proptosis
12	Liao *et al*.^19^	China Taiwan	Retrospective case series	pVEP	n.a.	22	—	30–76 (58.4)	DON	The differences in P100 (latency) before and after DON treatment
13	Wei *et al*.^20^	China Taiwan	Prospective case series	pVEP	n.a.	76	22–79 (46.7)	—	TAO	Correlation between P100 (latency) and VA, degree of proptosis, color test, visual field test, OCT and extraocular muscles measurements
14	Lipski *et al*.^21^	Germany	Retrospective case series	pVEP	✓2004^49^	15	—	43–76 (55)	DON	The differences in P100 (amplitude & latency) before and after DON treatment
15	Perez-Rico *et al*.^22^	Spain	Prospective case series	mfVEP	—	65	47.5 ± 11.5	—	TAO/Control	The difference in latency between TAO and control
16	Pawlowski *et al*.^23^	Poland	Prospective case-control study	pERG	✓2012^41^	21	24–55 (36)	—	TAO/Control	The difference in P50 amplitude between TAO and control

DON = dysthyroid optic neuropathy; EPS = electrophysiological studies; fVEP = flash visual evoked potential; ISCEV = International Society for the Clinical Electrophysiology of Vision Standard; mfVEP = multifocal visual evoked potential; n.a. = not available; OCT = optical coherence tomography; pERG = pattern electroretinography; pVEP = pattern visual evoked potential; TAO = thyroid associated orbitopathy; VA = visual acuity.

### Phenotypic definition of subjects

Study populations were phenotypically defined as patients with DON, TAO, or healthy subjects. Clinical features of DON included optic disc swelling, relative afferent pupillary defect, decreased visual acuity, impaired color vision, and visual field defect^31^. DON was considered “definite” if there was optic disc swelling or 2 of the four other clinical features above without alternative explanation in a patient with TAO^32^. Subclinical or “equivocal” DON was proposed by some as the presence of optic nerve dysfunction in TAO patients without the full-blown clinical features of DON^15^, often identified by abnormal electrophysiological changes^8,12,13,16,18,22^.

### Flash VEP (fVEP) in TAO & DON

Only 2 earlier studies reported use of fVEP in TAO and DON patients (Tables 2 and 3)^9,11^. Alteration in P2 amplitude was reported in clinically evident DON^11^. Tsaloumas *et al*. found significantly smaller P2 amplitude in DON eyes which improved either after orbital decompressions (6.83 ± 0.92 *vs*. 13.12 ± 1.65 µV; *P* < 0.05) or 2 weeks of high-dose systemic steroids (7.00 ± 1.10 *vs*. 9.61 ± 1.43 µV; *P* < 0.05^11^. However, treatment-related improvement was not shown in Setala’s study after decompression or radiotherapy^9^.

**Table 2:** Summary outcomes of observational case series and case-control studies on the use of VEP in DON/TAO.

No.	Author (year)	Age range (mean)			Sample size (eyes)			VEP outcome	Mean ± SD		
		DON	TAO	Control	DON	TAO	Control		DON	TAO	Control
**fVEP**											
4	Tsaloumas *et al*.^11^	26–73 (49.1)	23–68 (45.1)	22–68 (46.1)	8 (13)	15 (30)	20 (40)	P2 amplitude (µV)	6.83 ± 0.92^†‡^	12.40 ± 1.05	11.72 ± 1.16
								P2 latency (ms)	112.0 ± 4.46	110.1 ± 2.65	109.6 ± 2.08
**pVEP**											
1	Wijngaarde *et al*.^8^		n.a.	n.a.		33 (66)	20 (40)	P100 amplitude (µV)	n.a.	Data n.a.	n.a.
								P100 latency (ms)	n.a.	Data n.a^‡^	n.a.
3	Shawkat *et al*.^10^	37–62 (47.3)	37–62 (47.3)	37–62 (47.3)	10 (10)	10 (10)	10 (10)	P100 amplitude (µV)	11.9 ± 6.4^*^	21.2 ± 9.7	Data n.a.
								P100 latency (ms)	115.2 ± 5.7^*§^	110.3 ± 5.1	103.2 ± 4.3
4	Tsaloumas *et al*.^11^	26–73 (49.1)	23–68 (45.1)	22–68 (46.1)	8 (13)	15 (30)	20 (40)	P100 amplitude (µV)	3.67 ± 0.81^†§^	8.55 ± 0.73	8.97 ± 0.59
								P100 latency (ms)	129.2 ± 7.13^*‡^	111 ± 1.86	108.2 ± 1.19
5	Spadea *et al*.^12^		(57.2)	41–60		9 (18)	40 (40)	P100 amplitude (µV)	n.a.	3.47 ± 3.81^‡^	9.78 ± 4.26
								P100 latency (ms)	n.a.	126.7 ± 10.7 ‡	118.5 ± 5.7
6	Salvi *et al*.^13^		14–77 (45.3)	14–73 (41.8)		88 (172)	29 (56)	P100 amplitude (µV)	n.a.	10.2 ± 0.3	11.3 ± 0.6
								P100 latency (ms)	n.a.	105.6 ± 0.5^§^	102.0 ± 0.5
8	Rutecka-Debniak *et al*.^15^	18–74	18–74	18–74	12 (21)	13 (26)		N75 latency (ms)	90.0 ± 17.9^*^	80.3 ± 14.7	n.a.
								P100 latency (ms)	124.4 ± 15.4^*^	114.9 ± 11.2	n.a.
9	Acaroglu *et al*.^16^		20–65 (41.7)	23–65 (42.3)		16 (32)	15 (30)	P100 latency (ms)	n.a.	122.0 ± 14.4^§^	105.9 ± 7.7
10	Ambrosio *et al*.^17^	(42.5)		(44.3)	14 (28)		20 (40)	P100 amplitude (µV)	Data n.a^§^	n.a.	n.a.
								P100 latency (ms)	Data n.a^§^	n.a.	n.a.
11	Pawlowski *et al*.^18^		(35.6)	(28.6)		15 (30)	12 (24)	N75 latency (ms)	n.a.	79.0 ± 3.7^§^	73.9 ± 2.8
								P100 amplitude (µV)	n.a.	7.3 ± 3.5	6.5 ± 2.5
								P100 latency (ms)	n.a.	106.2 ± 4.4^‡^	102.4 ± 2.7
13	Wei *et al*.^20^		22–79 (46.7)			76 (151)		P100 latency (ms)	n.a.	103.7 ± 10.0	n.a.
**mfVEP**											
15	Perez-Rico *et al*.^22^		(47.5)	(48.1)		34 (65)	31 (62)	mfVEP latency (ms)	n.a.	6.57 ± 1.90 ‡	2.12 ± 1.72

DON = dysthyroid optic neuropathy; fVEP = flash visual evoked potential; mfVEP = multifocal visual evoked potential; ms = millisecond; n.a. = not available; pERG = pattern electroretinography; pVEP = pattern visual evoked potential; TAO = thyroid associated orbitopathy; VEP = visual evoked potential; µV = microvolts. *P < 0.05 compared to TAO without DON, ^†^ *P* < 0.001 compared to TAO without DON, ^‡^ *P* < 0.05 compared to Control, ^§^ *P* < 0.001 compared to Control.

**Table 3:** Summary outcomes of longitudinal case series comparing VEP changes before and after treatment for DON/TAO.

No.	Author (year)	Definition of cases	Age group	Sample size (eyes)	Treatment	VEP outcome	Reported values (mean ± SD)	
							Pre-treatment	Post-treatment
**fVEP**								
2	Setala *et al*.^9^	TAO	49–66 (55.8)	7 (13)	Decompression	N60 amplitude (µV)	15.8 ± 6.1	13.8 ± 6.9
						N60 latency (ms)	83.1 ± 21.6	81.9 ± 16.6
						P120 amplitude (µV)	8.0 ± 4.0	7.7 ± 4.2
						P120 latency (ms)	130.0 ± 21.3	129.4 ± 20.5
		TAO	50–64 (55.3)	3 (6)	Irradiation	N60 amplitude (µV)	16.8 ± 5.7	13.8 ± 6.0
						N60 latency (ms)	80.9 ± 7.0	87.5 ± 7.4
						P120 amplitude (µV)	8.5 ± 8.3	6.7 ± 7.3
						P120 latency (ms)	114.8 ± 14.8	123.2 ± 17.0
4	Tsaloumas *et al*.^11^	DON	26–73 (49.1)	6	Decompression	P2 amplitude (µV)	6.83 ± 0.92	13.12 ± 1.65*
						P2 latency (ms)	112.0 ± 4.46	106.7 ± 3.34
		DON	26–73 (49.1)	10	2 weeks High-dose steroids	P2 amplitude (µV)	7.00 ± 1.10	9.61 ± 1.43*
						P2 latency (ms)	118.4 ± 5.79	108.3 ± 5.47
**pVEP**								
4	Tsaloumas *et al*.^11^	DON	26–73 (49.1)	6	Decompression	P100 amplitude (µV)	3.67 ± 0.81	6.50 ± 0.67*
						P100 latency (ms)	129.2 ± 7.13	114.0 ± 4.47*
		DON	26–73 (49.1)	10	2 weeks High-dose steroids	P100 amplitude (µV)	5.30 ± 0.89	8.06 ± 0.80*
						P100 latency (ms)	116.1 ± 4.71	111.4 ± 4.89
8	Rutecka-Debniak *et al*.^15^	DON	18–74	12 (21)	Unspecified	N75 latency (ms)	93.3 ± 18.7	78.8 ± 7.7*
						P100 latency (ms)	126.0 ± 15.9	108.0 ± 5.3*
		TAO	18–74	13 (18)	Unspecified	N75 latency (ms)	81.7 ± 16.6	74.6 ± 7.9
						P100 latency (ms)	114.8 ± 12.6	107.3 ± 13.2*
12	Liao *et al*.^19^	DON	30–76 (58.4)	22 (38)	Decompression	P100 latency (ms)	134.8 ± 22.1	107.3 ± 4.0^†^
14	Lipski *et al*.^21^	DON	43–76 (55)	15 (30)	Decompression	P100 amplitude (µV)	4.45 ± 2.3	8.8 ± 6.32*
						P100 latency (ms)	130.2 ± 11.22	127.8 ± 12.07

DON = dysthyroid optic neuropathy; fVEP = flash visual evoked potential; ms = millisecond; No. = number; pVEP = pattern visual evoked potential; TAO = thyroid associated orbitopathy; VEP = visual evoked potential; µV = microvolts. *P < 0.05 compared to pre-treatment, ^†^ *P* < 0.001 compared to pre-treatment.

### Pattern VEP (pVEP) in TAO & DON

#### Comparison of pVEP results in DON, TAO, and normal controls

P100 latency, P100 amplitude, and N75 latency were compared between DON and normal controls in 3 studies^10,11,17^. An increase in P100 latency of patients with DON was reported by Shawkat *et al*. (115.2 ± 5.7 *vs*. 103.2 ± 4.3 ms, *P* = 0.0005)^10^, Tsaloumas *et al*. (129.2 ± 7.1 *vs*. 108.2 ± 1.2 ms, *P* < 0.005)^11^, and Ambrosio *et al*. (*P* < 0.0001)^17^. A decrease in P100 amplitude was found in eyes with DON compared to control by Tsaloumas *et al*. (3.67 ± 0.81 *vs*. 8.97 ± 0.59 µV, *P* < 0.001)^11^ and Ambrosio *et al*. (*P* < 0.0001)^17^.

Comparisons between TAO eyes with or without DON were reported in 3 studies^10,11,15^. Significant increases in P100 latency in eyes with DON were shown by Shawkat *et al*. (115.2 ± 5.7 *vs*. 110.3 ± 5.1 ms, *P* = 0.043)^10^, Tsaloumas *et al*. (129.2 ± 7.13 *vs*. 111 ± 1.86 ms, *P* < 0.005)^11^, and Rutecka-Debniak *et al*. (124.4 ± 15.4 *vs*. 114.9 ± 11.2 ms, *P* = 0.05)^15^. Significant decreases in P100 amplitude in DON patients were reported by Shawkat *et al*. (11.9 ± 6.4 *vs*. 21.2 ± 9.7 µV, *P* = 0.018)^10^ and Tsaloumas *et al*. (3.67 ± 0.81 *vs*. 8.55 ± 0.73 µV, *P* < 0.001)^11^. Moreover, the mean N75 latency of eyes with DON was also increased (90.0 ± 17.9 *vs*. 80.3 ± 14.7 ms, *P* = 0.01)^15^.

Five studies reported significant increases in P100 and N75 latencies comparing eyes from TAO patients without DON to healthy eyes (Table 2)^8,12,13,16,18^. Wijngaarde *et al*. first reported significant increase in P100 latency of TAO to healthy eyes (*P* < 0.01)^8^. Spadea *et al*. (126.7 ± 10.7 *vs*. 118.5 ± 5.7 ms, *P* < 0.05)^12^, Salvi *et al*. (105.6 ± 0.5 *vs*. 102.0 ± 0.5 ms, *P* < 0.001)^13^, Acaroglu *et al*. (122.0 ± 14.4 *vs*. 105.9 ± 7.7 ms, *P* = 0.0004)^16^, and Pawlowski *et al*. (106.2 ± 4.4 *vs*. 102.4 ± 2.7 ms, *P* < 0.01) also found increased P100 latencies in eyes from TAO subjects without clinical evidence of DON when compared with controls^18^. In addition, Pawlowski *et al*. found an increase in N75 latency (79.0 ± 3.7 *vs*. 73.9 ± 2.8 ms, *P* < 0.001)^19^, while Spadea *et al*. showed a decrease in P100 amplitude (3.47 ± 3.81 *vs*. 9.78 ± 4.26 µV, *P* < 0.05) in TAO patients comparing to normal subjects^12^. However, the differences between eyes from TAO patients and normal controls in N75 and P100 latencies were insignificant in other studies (Shawkat *et al*.^10^ and Tsaloumas *et al*.^11^). While these TAO patients did not show clinical evidence of DON, abnormal pVEP in particular prolonged P100 latencies may present electrophysiological evidence of early or subclinical optic nerve dysfunction in TAO patients.

### Correlation of pVEP latencies with clinical parameters

Four studies investigated correlation between pVEP latencies and clinical parameters (Table 4)^8,16,18,20^. Wijngaarde *et al*. reported a mild but significant correlation (r = 0.27, *P* value not available) of P100 latency with Snellen visual acuity^8^, while Wei *et al*. reported a similar degree of correlation without statistical significance (r = 0.278, *P* > 0.05) using LogMAR visual acuity^20^. In the latter study, correlation of P100 latency was moderate and statistically significant with total cross-sectional areas of all extraocular rectus muscles (EOM-A) (r = 0.496, *P* < 0.01); moderate but insignificant with ratio between the total cross-sectional area of all extraocular rectus muscles and the orbital area (r = 0.482, *P* > 0.05), mild and insignificant with total error of 100-hue color sensation (r = 0.363, *P* > 0.05) and with mean deviation of retinal sensitivity (MD) in perimetry (r = −0.342, *P* > 0.05). On the other hand, the correlation between peripapillary nerve fiber layer thickness and degree of exophthalmos with P100 latency was insignificant^20^. Acaroglu *et al*. reported a mild but significant correlation between the disease activity (clinical activity score) and P100 latency (r = 0.364, *P* = 0.04)^16^.

**Table 4:** Correlations between pVEP latencies and clinical measurements of DON/TAO.

No.	Author (year)	Definition of subjects	Age range (mean)	Sample size (eyes)	VEP latency	Clinical measurement	Correlation	
							*P* value	r
1	Wijngaarde *et al*.^8^	TAO	n/a	66	P100	VA	Significant	0.270
9	Acaroglu *et al*.^16^	TAO	20–65 (41.7)	32	P100	CAS	0.0406	0.364
11	Pawlowski *et al*.^18^	TAO	(35.6)	30	N75	Exophthalmos	<0.01	0.510
					P100	IOP	Insignificant	—
					P100	Exophthalmos	Insignificant	—
13	Wei *et al*.^20^	TAO	22–79 (46.7)	151	P100	logMAR	<0.1	0.278
					P100	Exophthalmos	Insignificant	−0.126
					P100	total error	<0.1	0.363
					P100	MD	<0.1	−0.342
					P100	ON	Insignificant	−0.055
					P100	M/O ratio	<0.1	0.482
					P100	EOM-A	<0.01	0.496

CAS = clinical activity score; DON = dysthyroid optic neuropathy; EOM-A = cross-sectional area of all extraocular rectus muscles; IOP = intraocular pressure; logMAR = logarithm of the minimal angle of resolution; MD = mean deviation of retinal sensitivity; M/O ratio = ratio between the cross-sectional area of all extraocular rectus muscles and the orbital area; No. = number; ON = peripapillary nerve fiber thickness; pVEP = pattern visual evoked potential; r = correlation coefficient; TAO = thyroid associated orbitopathy; total error = total error of 100-hue color sensation; VA = visual acuity; VEP = visual evoked potential.

The correlation between degree of exophthalmos and pVEP varied among studies. Pawlowski *et al*. reported a moderate and significant correlation between degree of proptosis and N75 latency (r = 0.51, *P* < 0.01) but not with p100 latency^18^. On the other hand, Wijngaarde *et al*. described a mild correlation coefficient between degree of proptosis and P100 latency (r and *P* value not available)^8^, while Wei *et al*. reported poor and insignificant correlation (r = −0.126, *P* value not available)^20^.

### pVEP after treatments

Four studies reported the pVEP results before and after treatments including high-dose steroids, orbital radiotherapy and/or decompression (Table 3)^11,15,19,21^. While treatment strategies varied, increase in p100 amplitude and/or decrease in p100 latency post-treatment were generally observed. More improvements were observed in eyes with DON than those without. Three studies reported more than 10% decrease in P100 latency after treatment of DON. Tsaloumas *et al*. reported a significant decrease (from 129.2 ± 7.13 to 114.0 ± 4.47 ms, *P* < 0.01)^11^, and so did Rutecka-Debniak *et al*. (from 126.0 ± 15.9 to 108.0 ± 5.3 ms, *P* = 0.01)^15^ and Liao *et al*. (from 134.8 ± 22.1 to 107.3 ± 4.0 ms, *P* < 0.001)^19^. Rutecka-Debniak *et al*. also reported a significant decrease in N75 latency in eyes with DON after treatment (from 93.3 ± 18.7 to 78.8 ± 7.7 ms, *P* = 0.01)^15^. Significant increase in P100 amplitude over 50% was reported by Tsaloumas *et al*. after decompression (from 3.67 ± 0.81 to 6.50 ± 0.67 µV, *P* < 0.01) and high-dose steroids treatment (from 5.30 ± 0.89 to 8.06 ± 0.80 µV, *P* < 0.01)^11^. Lipski *et al*. also reported significant increase in P100 amplitude after bony orbital decompression (from 4.45 ± 2.3 to 8.8 ± 6.32 µV, *P* < 0.05)^21^.

In TAO eyes with no clinical evidence of DON but prolonged P100 latency, Rutecka-Debniak *et al*. reported a significant decrease after treatment (from 114.8 ± 12.6 to 107.3 ± 13.2 ms, *P* = 0.05)^15^. There was no post-treatment change in TAO eyes with normal pre-treatment VEP.

### Multifocal VEP (mfVEP) in TAO

In 2012, Perez-Rico *et al*. first reported the use of mfVEP in TAO patients without DON^22^. There was a significant increase in mean latency in TAO group compared to age-matched control (2.12 ± 1.72 *vs*. 6.57 ± 1.90 ms, *P* < 0.05) and 23 eyes (35.4%) had abnormal mfVEP amplitude and/or latency. By interocular comparison, 12.3% of TAO eyes showed decreased amplitude and 13.8% of them showed increased latency. Visual acuity was significantly related to mfVEP amplitude changes (mean difference = −0.104, *P* = 0.018), while intraocular pressure measured at upgaze was significantly related to mfVEP latency changes (mean difference = 2.595, *P* = 0.028). No statistically significant relationship was observed between mfVEP parameters and standard automated perimetry results or nerve fiber layer thickness measured on optical coherence tomography^22^.

### Electroretinography (ERG) in TAO

Comparing TAO eyes with controls, Spadea *et al*. found significant decreases in amplitudes for both P50 (1.17 ± 0.58 *vs*. 1.74 ± 0.50 µV, *P* < 0.05) and N95 (1.71 ± 1.10 *vs*. 2.37 ± 0.59 µV, *P* < 0.05)^12^. No significant difference was found in latency^12^. Genovesi-Ebert *et al*. reported significantly smaller (*P* < 0.0001) pERG amplitude in TAO eyes without providing numerical results^14^. They also described a negative correlation of pERG amplitude with optic nerve diameter measured by ultrasonography. Pawlowski *et al*. reported significant decrease in P50 amplitude in TAO eyes (2.04 ± 0.99 *vs*. 2.69 ± 0.88 µV, *P* < 0.05) but not in N95 amplitude or latencies^23^. 3 studies reported drop in P50 amplitude^12,14,23^, with statistical significance shown by Spadea *et al*. and Pawlowski *et al*.^12,23^.

### Assessment of the quality of study and grading of clinical recommendation

The 12 studies on VEPs were assessed according to the NOS (Newcastle-Ottawa Scale) quality assessment of case-control studies^33^ (Table 5). The study with best quality was carried out by Tsaloumas *et al*. in 1994^11^. Clinical recommendation of EPS in detecting and monitoring visual dysfunction in TAO was rated according to the American Academy of Ophthalmology on preparing Preferred Practice Pattern (PPP) guidelines (Table 6)^34^. pVEP was given level A importance in application and level II in strength of evidence.

**Table 5:** Quality Assessment for Included Case-control Studies.

Author	Newcastle-Ottawa Quality Assessment Scale (NOS) for Case-control Study^33^									
	Selection				Comparability		Exposure			Total Stars
(Year of Publication)	Case Definition	Representativeness of Cases	Selection of Controls	Definition of Controls	Comparability of cases and controls (a)	Comparability of cases and controls (b)	Ascertainment of exposure	Same method of ascertainment	Non-Response rate	
Wijngaarde *et al*.^8^	−	−	−	−	−	−	−	*	n.a.	1
Shawkat *et al*.^10^	−	−	−	−	*	*	−	*	n.a.	3
Tsaloumas *et al*.^11^	*	*	*	*	*	*	−	*	n.a.	7
Spadea *et al*.^12^	*	−	−	*	*	−	−	*	n.a.	4
Salvi *et al*.^13^	−	*	−	*	*	−	−	*	n.a.	4
Genovesi-Ebert *et al*.^14^	−	−	−	−	*	−	−	*	n.a.	2
Rutecka-Debniak *et al*.^15^	−	−	−	*	−	−	−	*	n.a.	2
Acaroglu *et al*.^16^	*	−	−	*	*	*	−	*	n.a.	5
Ambrosio *et al*.^17^	*	−	−	*	*	−	−	*	n.a.	4
Pawlowski *et al*.^18^	*	−	*	*	*	*	−	*	n.a.	6
Perez-Rico *et al*.^22^	*	*	−	*	*	*	−	*	n.a.	6
Pawlowski *et al*.^23^	*	−	*	*	*	*	−	*	n.a.	6

*A star is awarded when the study meets the quality standard of an item. Details of the requirements of each item can be found in NOS for Case-control Study checklist^33^. n.a.: not available. Note: A study may be awarded a maximum of one star for each item within the Selection and Exposure categories. A maximum of two stars may be given for Comparability. A score of ≥7 stars is indicative of a high-quality study^33^.

**Table 6:** Clinical recommendation of VEP or ERG in detecting visual dysfunction in TAO.

Clinical care	Recommendation	Evidence rating
Detecting and monitoring visual dysfunction in TAO	The use of fVEP	[B:II]
Detecting and monitoring visual dysfunction in TAO	The use of pVEP	[A:II]
Detecting and monitoring visual dysfunction in TAO	The use of mfVEP	[B:II]
Detecting and monitoring visual dysfunction in TAO	The use of pERG	[C:II]

A = most important application; B = moderately important application; C = relevant but not critical application; II = well-designed cohort or case-control analytic studies, preferably from more than one center, or multiple-time series with or without the intervention.

## Discussion

Clinical features of DON may include impaired visual acuity and color vision, visual field, afferent and relative affect pupillary defect (APD/RAPD), optic disc hyperemia or swelling^5,31,35^. In practice, these features rarely co-exist while ocular co-morbidities often confound with clinical assessment^35^. The European Group on Graves’ Orbitopathy (EUGOGO) was the first to propose that the presence of optic disc swelling alone or any other two of the above abnormalities without an alternative explanation suggested the presence of DON in any TAO patient^35,36^. Among the 94 eyes recruited, impaired visual acuity (<20/40), color vision, visual field defects, relative afferent pupillary defect and optic disc swelling were present in only 73%, 77%, 71%, 45%, and 56% of eyes subsequently diagnosed to have “definite DON”. On the other hand, these abnormalities were also found in 32%, 7%, 13%, 0% and 5% of eyes subsequently diagnosed to have “no DON”. These results implied that none of the individual findings of optic nerve dysfunction was found to be sensitive or specific enough to diagnose or exclude DON. Proptosis or increased clinical activity scores (≥3/7) were absent in more than one-third of eyes with “definite” DON^35^. Despite its serious visual consequences, no widespread consensus on the diagnostic criteria of DON is available to date. The challenge in diagnosing DON at its early stage or in patients with ocular comorbidities remains.

Electrophysiological studies (EPS), including visual evoked potential (VEP) and electroretinogram (ERG) were adopted to provide objective evaluation and correlation with the presence and/or severity of DON. VEP refers to the electrophysiological signals extracted from visual cortex during visual stimulation over the retina^37^. Any disturbance along the visual pathway or visual cortex results in VEP abnormalities (decrease in amplitude or increase in latency). It was first reported in 1972 by Halliday *et al*. to assess optic neuritis^38^. Subsequently it was used in patients with DON in 1980 by Wijngaarde *et al*.^8^. Three types of VEP have been used: flash VEP (fVEP), pattern VEP (pVEP), and multifocal VEP (mfVEP) (Table 7). fVEP uses a diffuse flash stimulating the entire retina for a mass response. Therefore, localized abnormal response may be averaged out and left undetected. pVEP uses checkerboard pattern reversal simulation covering the central 15° visual field. The major components of pVEP are a large positive wave at peak latency of about 100 milliseconds (P100) and a negative wave peaking at 70 milliseconds (N70). Any delay in P100 latency or decrease in amplitude measured from N70 to P100 suggests the presence of optic neuropathy^37^. Since the first report on pVEP in assessing visual function in TAO patients by Wijngaarde *et al*. in ref.^8,9^ other studies were published comparing the use of pVEP in TAO patients with or without DON (Table 2). mfVEP records signals from multiple stimuli given simultaneously across 20° to 25° of the central visual field enabling assessment of small local defects^39^.

**Table 7:** Features of included studies.

EPS test	Key features	No. of studies	Reported parameters	Reference
**Visual evoked potential**				
fVEP	Diffuse flash stimulus, full-field, one response, examine whole visual pathway	2	amplitude & latency of P2, N60, P120	9,11
pVEP	Checkerboard pattern reversal stimulus, central≥15° field, one response, examine whole visual pathway	12	amplitude & latency of N75, P100	8,10–13,15–21
mfVEP	16 checks times 60 sectors stimulus, central 20 to 25° field, 60 topographic responses, examine whole visual pathway in 60 sectors	1	amplitude & latency	22
**Electroretinography**				
fERG	Diffuse flash stimulus, full-field, one response, examine retinal cells	0	n/a	n/a
pERG	Checkerboard pattern reversal stimulus, central 15° field, one response, examine retinal cells	3	amplitude & latency of N35-P50, P50-N95	12,14,23
mfERG	103 scaled hexagons stimulus, central 25° field, 103 topographic responses, examine retinal cells in 103 sectors	0	n/a	n/a

EPS = electrophysiological studies; fVEP = flash visual evoked potential; pVEP = pattern visual evoked potential; mfVEP = multifocal visual evoked potential; pERG = pattern electroretinography; mfERG = multifocal electroretinography; No. = number; n/a. = not applicable.

ERG records the electrical response of the retina upon light stimulation by various types of corneal electrodes. ERG is widely used in retinal disorders but rarely in TAO^40^. Pattern electroretinogram (pERG) uses reversing black and white checkerboard stimulus to collect signals from inner retina and indirectly measure retinal ganglion cell function. Commonly used parameters of pERG include a prominent positive wave at approximately 50 millisecond (P50) and a larger negative wave at about 95 millisecond (N95)^41^. pERG was used for evaluating early ganglion cell dysfunction in glaucoma patients since 1980s^42,43^. pERG alteration was also reported in animal models of optic nerve transection during retrograde degeneration of retinal ganglion cells^44,45^. In clinical practice, combined interpretation of pVEP and pERG helps to differentiate retinal (abnormal pVEP and pERG) from optic nerve disorders (abnormal pVEP and normal pERG)^46^.

Here we report the first systematic review on the use of EPS in DON. pVEP has been the most widely reported EPS in DON. Case-control studies reported significant differences of pVEP parameters among eyes with DON, TAO only and from controls^8,10–13,15–18,22^. Prolonged P100 latency was found comparing either eyes with DON to eyes without from TAO patients or eyes from TAO patients to control. P100 latency correlated with visual acuity, clinical activity score, color vision, visual field, and orbital imaging parameters^8,20^. Significant improvement in pVEPs were found in patients after successful treatment of DON^11,15,19,21^.

We acknowledge insufficient evidence to support the use of pVEP as part of the diagnostic criteria of DON due to its limited availability and inherent variability. To improve generalizability for meta-analysis, future studies should adopt testing protocols by the International Society for the Clinical Electrophysiology of Vision (ISCEV) standards^37,41,47–49^, include age and/or gender-specific reference ranges, post-treatment follow-up results and all clinical parameters recommended by the EUGOGO^5,31,35,37^. Longitudinal follow-up of pVEP on TAO patients with equivocal or early clinical features of DON may shed insight on the natural history, treatment response and clincal implication on the evolving entity of “subclinical” DON.

In conclusion, pVEP was the most studied EPS in DON. Latency and amplitude of P100 were shown to be promising for the diagnosis and monitoring of DON. Future studies on pVEP using standardized settings will be required to fully evaluate its diagnostic accuracy and clinical utility in the management of DON.

## Methods

### Literature search

Literature search was performed in MEDLINE, EMBASE, and the Cochrane databases via Ovid platform. We formulated sensitive search strategies using the Boolean logic and search terms with controlled vocabularies (Medical Subject Heading terms): (“thyroid associated” OR “endocrine” OR “dysthyroid” OR “Graves”) AND (“orbitopathy[ies]” OR “ophthalmopathy[ies]”) OR (“ophthalmic Graves’ disease”) in combination with “optic neuropathy(ies)” (Table 8). The search was supplemented by manual screening of the reference lists of the relevant articles and reviews. Language filter was not applied in the search. We identified records published from January 1^st^, 1977 to August 20^th^, 2017.

**Table 8:** Search strategies used in MEDLINE and EMBASE.

No.	Search terms
1	((thyroid associated or thyroid-associated) and (orbitopathy or orbitopathies or ophthalmopathy or ophthalmopathies)).mp.
2	(endocrine and (orbitopathy or orbitopathies or ophthalmopathy or ophthalmopathies or exophthalmos)).mp.
3	ophthalmic Graves disease.mp.
4	(thyroid and (orbitopathy or orbitopathies or ophthalmopathy or ophthalmopathies)).mp.
5	(Graves adj1 (orbitopathy or orbitopathies or ophthalmopathy or ophthalmopathies)).mp.
6	(dysthyroid and (orbitopathy or orbitopathies or ophthalmopathy or ophthalmopathies)).mp.
7	1 or 2 or 3 or 4 or 5 or 6
8	(optic adj1 (neuropathy or neuropathies)).mp.
9	(optic adj1 nerve adj1 (disease or disorder)).mp.
10	8 or 9
11	7 and 10
12	(dysthyroid adj1 (optic adj1 (neuropathy or neuropathies))).mp.
13	11 or 12

### Eligibility criteria

Studies were included in the systematic review according to the following criteria: (1) studies that used electrophysiological tests (e.g. VEP or ERG) to evaluate optic nerve dysfunction in patients with TAO or DON; and (2) studies can be observational case series, case-control study, cohort study, interventional case series, and clinical trials. Animal studies, case reports, reviews, abstracts, conference proceedings, and editorials were excluded.

### Assessment of the quality of study and level of evidence

NOS (Newcastle-Ottawa Scale)^33^ was adopted to evaluate the quality of the case-control studies. The clinical recommendation of VEP or ERG in detecting and monitoring visual dysfunction in TAO were rated from 2 aspects, “importance to the care process” and “the strength of evidence in the available literature”, according to the American Academy of Ophthalmology on preparing Preferred Practice Pattern (PPP) guidelines^34^. “Importance to the care process” represents the value of this application to improve the quality of the patient’s care in a meaningful way. Level A indicates the most important; level B indicates moderately important and level C indicates relevant but not critical application. “Strength of evidence” was rated in 3 levels. Level I includes evidence obtained from at least one properly conducted, well-designed, randomized, controlled trial. It also includes meta-analysis of randomized controlled trials. Level II includes well-designed controlled trials without randomization, well-designed cohort or case-control analytic studies, preferably from more than one center, or multiple-time series with or without the intervention. Level III includes evidence obtained from descriptive studies or case reports.
